# Peer Support and Psychosocial Pain Management Strategies for Children with Systemic Lupus Erythematosus

**DOI:** 10.1155/2015/238263

**Published:** 2015-10-25

**Authors:** Laura Nabors, Teminijesu John Ige, Bradley Fevrier

**Affiliations:** Health Promotion and Education Program, School of Human Services, University of Cincinnati, Cincinnati, OH 45221-0068, USA

## Abstract

This paper reviews information on Systemic Lupus Erythematosus (SLE) in children. Children with this chronic illness often experience pain related to their condition. They also can experience social isolation. This paper reviews psychosocial information on peer support and cognitive behavioral pain management strategies. The information presented in this paper provides new insights for health professionals assisting children and families in coping with psychological facets of this disease. Research focusing on ways by which peers and friends can support the child's use of psychological pain management strategies will provide new information for the literature.

## 1. Introduction

Children with Systemic Lupus Erythematosus (SLE) may experience chronic pain, cognitive dysfunction, and dermatological problems. SLE manifests in complex ways in children and can involve multiple organs [1]. There are many sources of pain for children with SLE, but some common ones are related to renal problems, abdominal pain, chest pain, and headaches [2]. SLE can impact the central nervous system, resulting in headaches, seizures, and cerebrovascular disease, all of which can result in increased pain experiences for children. Children with SLE may experience significant pain that goes unmanaged. In addition to physical complications, children with SLE can face a host of psychosocial issues. For example, children with SLE may experience depression and become isolated, which may have significant adverse effects on a child's interpersonal relationships [2–4]. The current paper reviews literature on peer relationships and psychosocial/cognitive behavioral interventions for pain management. Information presented in this paper allows health care professionals to view two key issues for children with SLE.

## 2. Prevalence and Incidence of SLE in Children

Variations exist in estimates of the incidence and prevalence of SLE [5, 6]. SLE is less common among children, as it is most often diagnosed in reproductive age in women. Approximately 0.36 to 1 per 100,000 children have SLE. This disease is more common in females and more severe for those in minority groups. SLE in children may be related to depression and other psychological symptoms that reduce quality of life [7–9].

## 3. Peer Relationships of Children with SLE

Peer relationships are vital in building self-esteem and a sense of acceptance and belonging, which are traits required for optimal psychosocial functioning [3, 10]. Peer support in healthcare can be defined as the provision of informational and emotional support by a peer who has acquired knowledge through experience and who possesses similar characteristics as the ill person [11]. Peer relationships are vital as children transition into adolescence where their sense of self determines how they gain independence, assume responsibility, and form successful interpersonal relationships [12]. Peer support can help to increase optimism and alleviate feelings of isolation and loneliness. This may assist children to better understand their illness and to learn and adopt healthy behaviors and disease management skills [11].

Children with SLE can experience challenges in building peer relationships due to experiencing isolation, low self-esteem, and coping with neurocognitive and affective disorders [2, 3]. Isolation that occurs within the context of chronic illness may lead to fewer friendships and lower levels of involvement in activities with peers [4, 13]. This situation may, in some cases, set the stage for social difficulties in adolescence and adulthood [14]. There is little information about interventions to improve peer support for children with SLE and little information about whether those with higher levels of peer support experience better functioning, reduced pain, and improved quality of life.

Research assessing peer support of children with chronic illnesses has shown that peer support can improve children's attitudes and functioning. For instance, in a randomized controlled trial of a “peer support” intervention for children with chronic illnesses, Rhee et al. found significant improvements in positive attitudes toward illness in an intervention group [15]. Also, McLaughlin et al. discovered that participating in a peer support social networking site led to improved social bonding in chronically ill children [16]. In their study, children who reported lacking support from others, poor family interactions, and low self-efficacy were more likely to use the social networking site. Consequently, when other types of support are low, social support from peers may be a resilience factor. In another study, Al-Sheyab and colleagues discovered significant improvements among participants of a peer-led education and support group in three areas: health-related quality of life, self-efficacy to resist negative health behaviors, and knowledge of best practices for illness management [17].

Health care professionals should inquire about social functioning for children with SLE and determine if a lack thereof is having a negative impact on emotional functioning and quality of life. In cases where peer support is low, several interventions may be considered. An intervention at the school level is to explain the child's medical condition to other children in the class. It is important to explain pain flares, missed school due to medical appointments, and differences in the child's behaviors while at school (e.g., riding the elevator to reduce walking) to peers in the child's class. When children have a better understanding of the nature of an illness it may be easier for them to respond appropriately to the needs of a classmate with SLE. If explaining to all peers in the classroom is not feasible, holding a meeting with the child's teacher, to explain how to educate peers on a more individual basis, may be appropriate. Involvement in extracurricular activities may afford opportunities to build social relationships. Requesting that parents monitor opportunities to engage in activities and build social opportunities is another important intervention if the child is coping with isolation or problems with emotional functioning. Finally, peers may not understand that the child with SLE has to cope with pain, take medicine, and use different pain management strategies. Carefully explaining pain flares and teaching peers about pain management may assist peers in understanding the illness and reduce any discomfort they have when interacting with the child.

## 4. Pain and Psychosocial Interventions for Pain for Children with SLE

Children with SLE often experience chronic pain and stiffness in joints, similar to that experienced by children with juvenile idiopathic arthritis. Experts in the field have cited pain amplification as a primary issue for children with SLE [2]. Medical treatments for joint pain include hydroxychloroquine and chloroquine. These medications reduce pain with relatively few adverse side effects. Methotrexate may be even more successful but produce greater side effects. Nonsteroidal anti-inflammatory drugs (NSAIDs) and corticosteroids are frontline treatments for joint pain. Mild exercise and omega-3 fatty acids are other possible treatments to relieve pain and fatigue [18].

Psychological strategies to manage pain may be useful as adjunctive interventions for treatment of SLE-related pain. This research team believes that many interventions used with adults can be applied to assist children and their families. Readers interested in learning more about psychosocial interventions are directed to a recent article by Barlow and her associates [19]. This paper presents a review of psychosocial strategies to assist adults. Before teaching the child pain management strategies, it may be advantageous to explain how pain works in the body using developmentally appropriate language. Emphasizing that pain is interpreted in one's brain and that how one thinks and feels can impact the pain experience is a key concept for children to grasp. This may help them to understand that what they think and do can help them manage their pain [19, 20].

Use of relaxation techniques and empowering imagery may help a child to reduce his or her focus on pain [21]. Relaxing scenes should be tailored to the child's own likes, and where one child might focus on going to the circus as a relaxing experience, another might find this too stimulating. The counselor or health professional typically works with the child to select relaxing experiences and then assists the child in recalling all of the details of a relaxing scene, including specific sights, sounds, smells, and details about the sequence of events. Imagery, such as picturing a superhero who is battling pain symptoms, can serve to distract the child, which pain experts agree is a powerful pain management strategy [20–22]. Children may also use positive self-talk to overcome self-defeating statements, such as “I am always hurting because I have SLE.” In contrast, the child can use positive self-statements, “I am feeling so happy and strong, and this makes me really strong to cope with my pain and feel better!” Teaching children that they can be problem solvers and use strategies to cope with their pain is a key message to boost children's self-efficacy for coping with pain [22, 23].

Relaxation strategies may help children cope with pain experiences [22]. Young children may have difficulty following complex relaxation strategies, but we have found that they are able to use a rock sponge activity, where they tense and relax their fist (provided this is not the area where pain symptoms are focused) to help them relax [21]. They also may tense and relax their legs or feet. We also work with children to talk about doing something fun, which may focus their thinking on something other than feelings of pain.

Some parents provide children with attention for pain behaviors, which accidentally reinforces pain complaints. Parents need to learn to reward their child for coping with his or her pain, adhering to doctors' recommendations, and following mild exercise regimes (being active can be a key pain management strategy). Involving children in physical exercise (as medically indicated) so that they become more active concomitantly reduces pain experiences [24]. Including strategies for parents, such as teaching parents to reinforce positive self-statements and ignore negative coping, should be emphasized. Research is lacking about whether age peers could help children with SLE by encouraging them to use pain management strategies. We believe that studies examining peers as supports in pain management efforts may be a fruitful area for research.

Degotardi and her colleagues evaluated a cognitive behavioral intervention for children with fibromyalgia [25]. The 8-week intervention consisted of education about the etiology of fibromyalgia; the role of stress and pain; how pain works in the body; and the relationship between emotional issues, sleep, and fatigue. Throughout the intervention (during multiple weeks), children learned to change negative thinking patterns and use positive thinking (such as reframing negative thoughts) to improve their functioning [25]. Children learned to set goals and were rewarded for changes in adaptive behaviors, in terms of managing their pain responses. Thus, goal setting, positive self-statements, changing negative thought patterns and self-talk, and use of distraction and relaxation were key strategies in the intervention package. Children in elementary school were included in the sample and it is noteworthy that interventions were tailored to key developmental issues for young children. Results were positive, indicating that children reported feeling more “in control” of their illness, felt less fatigue, and reported fewer disease-related symptoms. Parents reported that children's pain complaints lessened; children reported fewer somatic symptoms.

Walco and colleagues also used cognitive behavioral strategies to teach pain management strategies to 13 children with arthritis [23]. Pain management strategies included progressive muscle relaxation (tensing and relaxing muscle groups), meditative breathing, and guided imagery. Guided imagery occurs when an adult guides the child through imagining a relaxing scene, such as going to the beach or mountains. The guided imagery strategy in Walco et al.'s study served to distract children from thinking about pain [23]. Children can also generate positive images. An example might be a superhero, with superpowers to fight pain. This hero might have superpowers to “blow out” the hot fire of a pain experience. Parents who participated in the study were taught to reward coping behaviors and decrease attention paid to pain behaviors [23]. Future studies need to be conducted to determine if CBT strategies also are effective in reducing pain for children with SLE.

Brown et al. conducted a randomized controlled trial to deliver cognitive behavioral therapy to female adolescents with SLE [26]. Adolescents in this study were randomly assigned to receive health education, CBT, or be in a control group. The researchers had an impressive battery of assessment instruments to examine pain, psychosocial characteristics of participants, and quality of life. Those adolescents in the CBT group learned calming self-statements and techniques to diminish negative self-statements and affectivity associated with pain. Relaxation (progressive muscle relaxation, breathing), distraction (e.g., mental counting techniques), and problem-solving techniques were discussed with participants. Those in the education group learned about SLE and the importance of living a healthy lifestyle. Unfortunately, results of this study were not conclusive. Thus, reduced pain and improved quality of life were not discovered for the CBT group compared to the other two groups. However, the coping abilities of adolescents in the CBT group were significantly more advanced than those of females in the other two groups. The authors speculated that the dose of the intervention may not have been strong enough to produce intended results [26].

Our literature search also revealed studies using CBT interventions with adults. The interventions in these studies could also be used with children. Liang et al. conducted a systematic review of the literature investigating psychological interventions for adults with SLE [27]. They discovered six studies that were randomized controlled trials. After reviewing these studies, Liang et al. concluded that the psychological interventions were successful in reducing depression and improving the health status of participants with SLE. Two of the studies reviewed by Liang et al. presented information on CBT. These projects were conducted by Navarrete-Navarrete and colleagues and both presented valuable information on CBT [28, 29].

One study appeared particularly germane to the current paper. In this paper, Navarrete-Navarrete et al. discussed a CBT intervention for adults [28]. They randomly assigned 18 adults with SLE to a treatment group and 16 to a conventional care group. Those assigned to the treatment group participated in psychoeducational sessions. Participants learned CBT techniques including challenging and stopping negative thoughts, deep breathing for relaxation, and muscle relaxation. Participants in the treatment group reported higher quality of life after intervention and at a 15-month follow-up [28]. We believe the CBT techniques used for this project are easily transferable to children. For instance, children can be taught deep breathing through exercises with blowing bubbles. They can also learn to take slower breaths by slowly counting to three while inhaling, holding the breath (for a count of 3), and then slowly exhaling while counting to three. Children can learn to identify negative thoughts and replace negative thinking with positive statements. Finally, role-play or modeling can be used to teach children a thought-stopping exercise. Children can learn to identify a negative thought pattern, see a stop sign (to stop the negative thought), and then replace the negative thought with a positive image (e.g., going to a birthday party or an amusement park).

In another study with adults, Williams et al. developed an intervention to decrease stress in lupus patients [30]. Patients who were enrolled in the experimental group were involved in six weekly lessons drawn from the “Better Choice Better Health” Program developed by Stanford University [31, 32]. Meetings were facilitated by “nonhealth” professionals who also had a chronic illness. Lessons covered nutrition; ideas for coping with pain, fatigue, and isolation; improving activity; and communicating effectively with others about illness. Results indicated improved health and reduced experiences of pain, stress, and depression. Feelings of self-efficacy for disease management improved, which can have a positive impact on adherence to the treatment regimen. The authors concluded that the intervention was successful but that further studies will be important to understand the impact of individual components of the intervention program. We believe that many aspects of this program, such as discussing healthy living, coping with pain and fatigue, and ideas for improving activity, could be used to educate children and their parents.

## 5. Conclusions

This paper has highlighted the importance of peer support and use of CBT interventions to manage pain as two potential interventions to enhance resilience for children with SLE. Medical professionals need to implement and study resilience in children with SLE to determine paths of success for these children as they cope with a serious chronic illness. Further research examining psychological interventions in combination with concurrent medication therapies in improving pain and quality of life is needed. Our literature review did not yield studies assessing whether peers can support children with SLE in pain management efforts. Future studies examining the influence of peer support and CBT strategies for pain management are needed. Studies examining the influence of each of the aforementioned variables will determine if these factors impact child quality of life, emotional functioning, and social development.
